# Azacitidine in patients with WHO-defined AML – Results of 155 patients from the Austrian Azacitidine Registry of the AGMT-Study Group

**DOI:** 10.1186/1756-8722-6-32

**Published:** 2013-04-29

**Authors:** Lisa Pleyer, Reinhard Stauder, Sonja Burgstaller, Martin Schreder, Christoph Tinchon, Michael Pfeilstocker, Susanne Steinkirchner, Thomas Melchardt, Martina Mitrovic, Michael Girschikofsky, Alois Lang, Peter Krippl, Thamer Sliwa, Alexander Egle, Werner Linkesch, Daniela Voskova, Hubert Angermann, Richard Greil

**Affiliations:** 1 3rd Medical Department with Hematology and Medical Oncology, Hemostaseology, Rheumatology and Infectious Diseases, Laboratory for Immunological and Molecular Cancer Research, Oncologic Center, Paracelsus Medical University Hospital Salzburg, Müllnerhauptstrasse 48, Salzburg, 5020, Austria; 2 Internal Medicine V (Hematology and Oncology), Innsbruck Medical University, Anichstrasse 35, Innsbruck, 6020, Austria; 3 Department for Internal Medicine IV, Hospital Wels-Grieskirchen, Grieskirchnerstrasse 42, Wels, 4600, Austria; 4 1st Department of Internal Medicine, Center for Oncology and Hematology, Wilhelminenspital, Montleartstrasse 37, Vienna, 1160, Austria; 5 Department for Hematology and Oncology, LKH Leoben-Eisenerz, Radmeisterstrasse 7-9, Eisenerz, 8790, Austria; 6 IIIrd Medical Department, Hanusch Hospital, Heinrich-Collin-Strasse 30, Vienna, 1140, Austria; 7 1st Medical Department with Hematology, Stem cell transplantation, Hemostatsis and Medical Oncology, Elisabethinen Hospital, Fadingerstrasse 1, Linz, 4010, Austria; 8 Internal Medicine, Hospital Feldkirch, Carinagasse 47, Feldkirch, 6800, Austria; 9 Department for Internal Medicine, LKH Fuerstenfeld, Krankenhausgasse 1, Fuerstenfeld, 8280, Austria; 10 5th Medical Department with Oncology und Palliative Medicine, Hietzing, Wolkersbergenstrasse 1, Vienna, 1130, Austria; 11 Department of Hematology, Medical University, Auenbruggerplatz 1, Graz, 8036, Austria; 12 Internal Medicine 3, Center for Hematology and Medical Oncology, General Hospital-Linz GesmbH, Krankenhausstrasse 9, Linz, 4021, Austria; 13 UNIDATA GEODESIGN GmbH, Gaertnergasse 3, Vienna, 1030, Austria

**Keywords:** Austrian azacitidine registry, Azacitidine, AML, Overall survival, Prognostic factors, Bone marrow blasts

## Abstract

**Objective:**

The Austrian Azacitidine Registry is a multi-center database (ClinicalTrials.gov: NCT01595295). The nature and intent of the registry was to gain a comprehensive view of the use, safety and efficacy of the drug in a broad range of AML-patients treated in real-life scenarios.

**Patients and methods:**

The sole inclusion criteria were the diagnosis of WHO-AML and treatment with at least one dose of azacitidine. No formal exclusion criteria existed. A total of 155 AML-patients who were mostly unfit/ineligible for intensive chemotherapy, or had progressed despite conventional treatment, were included. True ITT-analyses and exploratory analyses regarding the potential prognostic value of baseline-variables/performance-/comorbidity-/risk-scores on overall survival (OS), were performed.

**Results:**

In this cohort of 155 pretreated (60%), and/or comorbid (87%), elderly (45% ≥75 years) AML-patients, azacitidine was well tolerated and efficacious, with an overall response rate (CR, mCR, PR, HI) of 45% in the total cohort (ITT) and 65% in patients evaluable according to IWG-criteria, respectively. Pre-treatment with conventional chemotherapy (*P = .113*), age ≤/>80 years (*P = .853*), number of comorbidities (*P = .476*), and bone marrow (BM) blast count (*P = .663*) did not influence OS. In multivariate analysis hematologic improvement alone (without the requirement of concomitant bone marrow blast reduction), although currently not regarded as a standard form of response assessment in AML, was sufficient to confer OS benefit (18.9 vs. 6.0 months; *P = .0015*). Further deepening of response after first response was associated with improved OS (24.7 vs. 13.7 months; *P < .001*).

**Conclusions:**

In this large cohort of AML-patients treated with azacitidine, age >80 years, number of comorbidities and/or BM-blasts >30% did not adversely impact OS.

## Background

Acute myeloid leukemia (AML) is an aggressive disease with an unfavorable prognosis [1,2]. Only approximately 1/3 of elderly AML-patients are eligible for intensive chemotherapeutic approaches. Results remain poor and unsatisfactory, even for those who do meet inclusion criteria for such treatment (induction mortality of up to 29%; 2 year OS 6-11%) [3-5]. Patients not suitable for intensive chemotherapy due to high age, comorbidities, poor performance status and/or adverse cytogenetics, or who have failed these treatment options, are frequently offered best supportive care (BSC) only [6], and the prognosis is dismal [7]. Other treatment options such as low-dose cytarabine, topotecan, gemtuzumab ozogamicin, clofarabine, cloretazine, or tipifarnib have not been promising [8-14]. Treatment options for AML-patients with >30% BM-blasts unfit/ineligible for intensive chemotherapeutic approaches are particularly limited, as hypomethylating agents were not approved for this patient group. Only very recently has decitabine been granted EMA-, but not FDA-approval for the treatment of WHO-AML patients aged 65 or older who are not candidates for standard induction chemotherapy (http://www.ema.europa.eu/ema/index.jsp?curl=pages/medicines/human/medicines/002221/human_med_001589.jsp&mid=WC0b01ac058001d124).

Azacitidine (Vidaza®) received FDA-approval for MDS and AML with 20-30% BM-blasts in 2004 based on the results of several CALGB-trials [15]. EMA-approval for HR-MDS and AML with 20-30% BM-blasts was granted in 2008 following the pivotal AZA-001 trial [16]. In a subset-analysis, azacitidine was shown to prolong OS in elderly AML-patients with 20-30% BM-blasts [17].

The Austrian Azacitidine Registry (AAR) (ClinicalTrials.gov: NCT01595295) comprises 155 AML-patients who did not qualify for intensive chemotherapeutic approaches in most cases, and is currently the largest cohort of AML-patients treated with azacitidine published to date, with the highest per capita coverage of AML-patients in a nationwide registry, suggesting limited selection (Additional file 1: Tables S1 and S2). The aim was to include most AML-patients treated with azacitidine in Austria, and to gain a comprehensive view of the use, safety and efficacy of the drug in a broad range of WHO-defined AML, including patients with >30% bone marrow blasts, who have limited other therapeutic options, and for whom the drug has not been approved yet.

## Results

### Patient characteristics

Between 02/2009 and 02/2012, 155 AML-patients from 12 specialized centers for hematology and medical oncology were included; No patients were excluded from the analyses (Additional file 2: Figure S1A). Patient baseline characteristics can be taken from Table 1. Median age was 73 (range 33–91); 57% of patients were older than 75, 23% were older than 80 and 8% were older than 85 years, respectively; 98 patients (63%) had >30% BM-blasts; 17% had an unfavorable karyotype, and 74% had an intermediate karyotype according to MRC-criteria [18]. In the absence of consensus for cytogenetic classification of AML in the elderly, we additionally assessed the IPSS cytogenetic risk categories (Table 1).

**Table 1:** Baseline characteristics

	
**Median age, years (range)**	73 (33–91)
WHO diagnosis*, n (%)	
t-AML	16 (10.3)
AML-RCA	16 (10.3)
AML-MRF only	88 (56.8)
AML-MRC	13 (8.4)
Preexisting MDS/MPN or MLD	58 (37.4)
Preexisting MDS/MPN or MLD + MRC	17 (11.0)
AML-NOS	35 (22.6)
**Peripheral blood blasts, n (%)**	
0%	58 (37.4)
> 0%	97 (62.6)
Mean,%	14
Median (range),%	3 (0–90)
**Bone marrow blasts, n (%)**	
<20%†	26 (16.8)
20–30%	31 (20.0)
>30% (off label use)	98 (63.2)
Mean,%	42
Median (range),%	35 (0–98)
**White blood cell count, n (%)**	
Non-MP-AML (< 10 G/l)	122 (78.7)
MP-AML (> 10 G/l)	33 (21.3)
**Transfusion dependence (TD), n (%)**	
Any type of TD	101 (65.2)
RBC-TD	97 (62.6)
PLT-TD	60 (38.7)
RBC-TD + PLT-TD	56 (36.1)
**IPSS cytogenetic risk**‡**, n (%)**	
Not evaluable	11 (7.1)
Good	91 (58.7)
Intermediate	26 (16.1)
Poor	27 (17.4)
**MRC cytogenetic risk**‡**, n (%)**	
Not evaluable	11 (7.1)
Good	3 (1.9)
Intermediate	115 (74.2)
High	26 (16.8)
**Comorbidities, n (%)**	
Thromboembolic episodes	21 (13.5)
Renal insufficiency	29 (18.7)
Liver disease	20 (12.9)
Diabetes mellitus	26 (16.8)
Coronary artery disease	34 (21.9)
COPD	15 (9.7)
Prior/concomitant malignancies	35 (22.6)
**Number of comorbidities, n (%)**	
0–1	66 (42.6)
2–3	61 (39.4)
> 3	28 (18.1)
**ECOG Prognostic Score, n (%)**	
ECOG <2	114 (73.6)
ECOG ≥2	41 (26.4)
**HCT-CI, n (%)**	
Low risk	46 (29.7)
Int. risk	46 (29.7)
High risk	50 (32.2)
No data	13 (8.4)
**Treatment prior to azacitidine**§**, n (%)**	
None	63 (40.6)
Erythropoietin stimulating agents	15 (9.7)
G-CSF	19 (12.3)
Thrombopoietin stimulating agents	1 (0.7)
Iron chelation therapy	5 (3.2)
Thalidomide	3 (1.9)
Lenalidomide	6 (3.9)
Low-dose cytarabine	10 (6.5)
Intensive chemotherapy	60 (38.7)
Others	16 (10.3)
**Reason for treatment, n (%)**	
1st line treatment	63 (40.6)
Bridging to allogeneic SCT^#^	4 (2.6)
Maintenance after CR to conventional chemotherapy	6 (3.9)
No CR to/early relapse after conventional chemotherapy	45 (29.0)
No CR to/early relapse after allogeneic SCT	5 (3.2)
No CR to other prior treatment	32 (20.6)

t-AML indicates treatment related AML; AML-RCA, AML with recurrent cytogenetic abnormalities; AML-MRF, AML with MDS related features; MPN, myeloproliferative neoplasia; MLD, multilineage dysplasia; MRC, MDS-related cytogenetics; AML-NOS, AML not otherwise specified; MP, myeloproliferative; COPD, chronic obstructive pulmonary disease; NHL, non-Hodgkin’s lymphoma; ECOG, Eastern Cooperative Oncology Group; CR, complete response; SCT, stem cell transplantation;

*If a patient fulfilled criteria for more than one WHO-category, weighting was performed as follows: t-AML > AML-RCA > AML-MRF.

†BM-blast count was <20%, in those patients with established AML who were refractory to, -or had no CR after-, conventional chemotherapy or allogeneic stem cell transplantation.

‡Pre-treatment cytogenetics were available in 92% of patients and were determined by conventional metaphase karyotyping (46%), interphase-FISH (16%), or both (38%).

§Numbers may add up to >100% as multiple selections were possible.

^#^None of the patients for whom azacitidine was intended as bridging was in complete remission to prior therapies, and all patients had relapsed after multiple lines of intensive chemotherapy; only one of these patients proceeded to allogeneic SCT (the others died whilst still on, or within 8 weeks after termination of azacitidine treatment due to disease progression and/or infectious complications).

### Treatment modalities

Azacitidine was administered as first line therapy in 41% of patients. Bridging to allogeneic stem cell transplantation (allo-SCT), maintenance after complete response (CR) to chemotherapy, no CR to, or early relapse after allo-SCT or conventional chemotherapy was the reason for treatment in 3%, 4%, 3% and 29% of patients, respectively (Table 1). Azacitidine was not always second line therapy in chemotherapy refractory patients, but also third, fourth, fifth or last line therapy for a relevant proportion of our patients (28%).

Azacitidine dose, application route, and administration schedule were exclusively based on the risk/benefit-estimation of the treating physician. The median and mean number, as well as range of azacitidine courses was 4, 6.3 and 1–24, respectively. Most cycles (90%) were applied subcutaneously (average dose/cycle 814 mg); 10% were given intravenously (average dose/cycle 870 mg); 79% of patients predominantly received 7 days of azacitidine (57% FDA-approved d1-7 (median dose/cycle 924 mg), 22% 5-2-2 (median dose/cycle 900 mg)) (Additional file 1: Table S3). FDA-approved azacitidine target-dose (75 mg/m^2^×7 +/-10%) was reached in 53% of applied cycles; 214/958 (22%) of all cycles were administered as ‘flat’ dosage (i.e. 100 mg azacitidine/cycle-day; median dose/cycle 700 mg). Dose reduction of azacitidine due to an adverse event (AE) was necessary in 28 (18%) patients.

### Concomitant treatment and best supportive care measures

In all 958 azacitidine cycles applied to AML-patients, erythropoietin stimulating agents (ESA) (3%), iron chelation treatment (ICT) (3%), and G-CSF (21%) were given in parallel to azacitidine when deemed necessary by the treating physician. ESA and ICT were rarely required after cycle 5, whereas G-CSF-usage did not show the same decline.

### Response

Overall response, defined as complete response (CR), marrow response (mCR), partial response (PR) and hematologic improvement (HI), was documented in 45.2% of the total intention-to-treat (ITT) cohort and in 65.4% of patients evaluable according to IWG-criteria [19] (i.e. had received >2 cycles of azacitidine); Hematologic improvement was documented in 32% (ITT) and 46% (IWG), respectively; Best marrow response [20] is shown in Table 2.

**Table 2:** **Response to azacitidine **[18]**,**[19]

	
Transfusion independence, n, (% ITT*), [% IWG†**]**	
PLT-TI	24/60 (40.0), 24/43 [55.8]
RBC-TI	35/97 (36.1), 35/69 [50.7]
**Hematologic improvement, n, (% ITT), [% IWG**‡**]**	
No HI	58 (37.4) [54.2]
HI-Any	49 (31.6) [45.8]
**Best marrow response, n (% ITT) [% eval.**§]	
CR	15 (9.7) [20.0]
mCR	5 (3.2) [6.7]
PR	32 (20.6) [42.7]
mSD	19 (12.3) [25.3]
Primary PD	4 (2.6) [5.3]
**Overall response, n, (% ITT), [% IWG**‡**]**	70 (45.2) [65.4]
CR	15 (9.7) [14.0]
mCR	5 (3.2) [4.7]
PR	32 (20.6) [29.9]
mSD with HI	4 (2.6) [3.7]
HI only	14 (9.0) [13.1]
No response	61 (39.4), 37 [34.6]

IWG indicates International Working Group Criteria; TI, transfusion independence; HI, hematologic improvement; mCR, marrow CR; mSD, marrow stable disease; BMP, bone marrow puncture; PD, progressive disease;

*Concerns all patients that were transfusion dependent at baseline;

†Concerns number of patients that were transfusion dependent at baseline and received >2 cycles of azacitidine and were thus evaluable for response assessment according to IWG-criteria;

‡Evaluable according to IWG-criteria, i.e. patients that received >2 cycles of azacitidine (n = 107);

§Concerns patients in whom bone marrow puncture was performed (n = 75); bone marrow assessment was performed as clinically necessary, and at the discretion of the respective treating physician; a total of 140 bone marrow punctures were performed in 75 patients (of patients in whom no bone marrow puncture was performed, 30 received only one and 18 received only two cycles of azacitidine; of these, 26 died within 2 months and a further 11 died within 6 months after stop of azacitidine treatment).

Taking a closer look at responding patients (n = 70), 77% received 7 days of azacitidine in the first cycle (65% d1-7, 13% 5-2-2) and 82% predominantly received 7 days of azacitidine over all cycles (52% d1-7 (median dose/cycle 871 mg), 30% 5-2-2 (median dose/cycle 837 mg) (Additional file 1: Table S3). Of note, the distribution of applied schedules, as well as the median and mean azacitidine dosages/cycle did not differ between responders and non-responders (Additional file 1: Table S3). The median and mean number, as well as range of cycles received by responding patients is 9, 10.3 and 1–31, respectively.

**Table 3:** Specific adverse events*,†

**Variable**	**Grade**	**n pts., (%)**	**n total events**
**Hematologic toxicity**^**#**^	G3–4	69 (44.5)	148
Thrombopenia	G3–4	38 (24.5)	87
Neutropenia	G3–4	49 (31.6)	99
Anemia	G3–4	33 (21.3)	80
**Bleeding events**	–	14 (9.0)	31
**Febrile neutropenia**	–	28 (18.1)	46
**Infectious complications**	Total	98 (63.2)	256
	G1	14 (9.0)	85
	G2	39 (25.2)	107
	G3	16 (10.3)	26
	G4	29 (18.7)	38
**Non-hematologic toxicity**			
Liver	G1-2	1 (0.6)	1
	G3-4	0 (0.0)	0
Kidney	G1-2	5 (3.2)	5
	G3-4	0 (0.0)	0
Heart	G1-2	3 (1.9)	5
	G3-4	13 (8.4)	15
Blood pressure	G1-2	2 (1.3)	2
	G3-4	1 (0.6)	1
Metabolic	G1-2	1 (0.6)	1
	G3-4	0(0.0)	0
Thromboembolic	G1-2	9 (5.8)	10
	G3-4	1 (0.6)	1
Neurologic	G1-2	11 (7.1)	18
	G3-4	1 (0.6)	1
Nausea	G1-2	16 (10.3)	24
	G3-4	0 (0.0)	0
Vomiting	G1-2	4 (2.6)	5
	G3-4	0 (0.0)	0
Constipation	G1-2	3 (1.9)	5
	G3-4	0 (0.0)	0
Diarrhea	G1-2	17 (11.0)	25
	G3-4	0 (0.0)	0
GIT-others	G1-2	11 (7.1)	11
	G3-4	0 (0.0)	0
Injection site reaction	G1-2	32 (20.6)	48
	G3-4	0 (0.0)	0
Fatigue	Total	65 (41.9)	99
	Relieved by rest	24 (15.5)	50
	Not relieved by rest	25 (16.1)	32
	Limiting self care	16 (10.3)	17
Pain	Total	46 (29.7)	78
	Mild	25 (16.1)	52
	Moderate	18 (11.6)	23
	Severe	3 (1.9)	3
Surgery	Total	20 (12.9)	24
	Elective	13 (8.4)	16
	Emergency	7 (4.5)	8
Fall	Total	14 (9.0)	16
	With fracture	8 (5.2)	9
	With hemorrhage	5 (3.2)	8
Novel solid tumor	Yes	3 (1.9)	3

*http://evs.nci.nih.gov/ftp1/CTCAE/About.html.

†National Cancer Institution Toxicity Criteria (http://ctep.cancer.gov/protocolDevelopment/electronic_applications/ctc.htm).

^**#**^Grade 3–4 cytopenias reported, are those that were documented as adverse events, and thus felt to be a worsening of pre-existing cytopenia by the respective treating physicians.

### Time to response and response deepening

Median (mean) time to first response was 4 (3.5) months. First response occurred after 3, 4 and 5 cycles in 44%, 77% and 87% of responding patients, respectively, but could be observed as late as cycle 10. First response was best response in 46/70 patients (66%). Further deepening of response after first response was seen in 24/70 (34%) of responders. Best response was reached by cycle 8 in 93%, but could be observed as late as cycle 19. Median (mean) time from first to best response was 2.8 (3.6) months, respectively.

### Toxicity and adverse events

A total of 501 adverse events were documented in 958 azacitidine cycles. The number of adverse events declined continuously from ~40% of patients in the 1^st^ cycle to <20% as of cycle ≥10. Overall, 32% of all adverse events and 20% of grade 3–4 adverse events were attributed to azacitidine; 22% resulted in hospitalization, 6% resulted in death; 69% had no consequence for azacitidine treatment; 6%, 14%, 9%, and 1% resulted in dose reduction, treatment pause, termination of treatment, or prolongation of azacitidine cycle duration >28 days, respectively.

Grade 3–4 neutropenia, thrombocytopenia, and anemia were documented in 32%, 25%, and 21% of patients, respectively; clinically relevant bleeding events were noted in 9% (Table 3). Non-hematologic toxicity was usually mild, the most common adverse events being fatigue (42%), unspecified pain (30%), gastro-intestinal (26%), and injection site reactions (21%). Infectious complications of any grade were documented in 63%, febrile neutropenia in 18% (Table 3). Grade 3–4 events occurred in 29% and were dominated by pulmonary infections, sepsis, and CMV-reactivations. Hospital admission was required in 43% and transfer to an intensive care unit was necessary in 4%.

Only 18 non-hematologic grade 3–4 events occurred in 16 patients (10%): 15/18 of these grade 3–4 events occurred in the cardiac system: left-ventricular output failure (n = 10), arrhythmia (n = 4), cardiac ischemia (n = 1). In 10/15 (67%) patients experiencing cardiac grade 3–4 events, pre-existing coronary artery disease, reduced cardiac function or arrhythmias were documented prior to azacitidine treatment, and worsening was not thought to be azacitidine-related.

### Overall survival and potential prognostic parameters

Median OS was 16.3 (95% CI 12.23–19.97) months as of first diagnosis, and 9.8 (95% CI 8.59–10.93) months as of treatment start with azacitidine. Median (mean) time from first diagnosis to treatment start with azacitidine for untreated (n = 63) versus pretreated (n = 92) patients was 0.6 (4.1) and 8.5 (14.9) months, respectively. Median (mean) time from initial diagnosis to azacitidine treatment for patients who received no, one, or multiple lines of conventional chemotherapy prior to azacitidine, was 0.7 (6.7), 1.8 (4.1), and 13.3 (21.7) months, respectively. 48 patients received ≤2 cycles of azacitidine; 23 of these died within 1 month of treatment termination, and a further 14 died within 6 months. Median (mean) time from azacitidine treatment stop to death was 1.8 (4.0) months, respectively.

**Figure 1:**
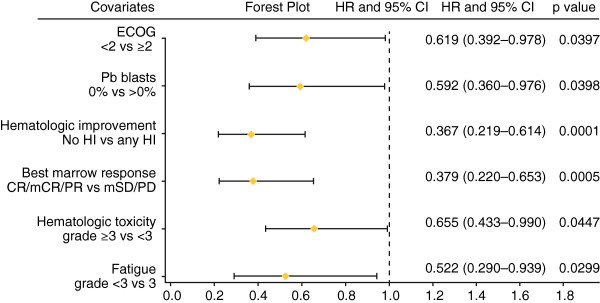
Factors significantly influencing overall survival in multivariate analysis.

In multivariate analysis the following baseline parameters had a significant effect on overall survival: peripheral blood blasts (*P = 0.0398*) and ECOG performance score (ECOG-PS) (*P = 0.0397*) (Figure 1 and Additional file 1: Table S5). In univariate analysis patients with adverse cytogenetics (-7q, -7, abn (3q), complex karyotype) (n = 28) had worse OS than patients with other cytogenetic abnormalities or normal karyotype (n = 115) (5.1 vs. 10.5 months, *P = 0.009*) (Additional file 1: Table S5). Patients pre-treated with ‘imids’ (n = 9), i.e. thalidomide or lenalidomide, may have worse OS: prior treatment with either of these substances was associated with a significant adverse effect on survival (P = 0.008, median OS 3.0 vs. 9.7 months, respectively) (Additional file 1: Table S5). However, the number of patients pretreated with thalidomide/lenalidomide was too small to establish strong conclusions or to perform multivariate analysis. Of the 6 patients pretreated with lenalidomide, 2 had preceeding MDS with -5q as sole aberration, one had a complex karyotype including -5q, one had a complex karyotype including monosomy 5, one had a normal karyotype, and in one patient karyotype was not evaluable.

The absolute number of comorbidities did not adversely affect OS of AML-patients treated with azacitidine with a cut-off of <3/≥3 comorbidities (*P = 0.151*). When a cut-off of <4/≥4 comorbidities was analyzed, there was trend for reduced OS, which was however not statistically significant (*P = 0.086*) (Additional file 1: Table S4). MRC-cytogenetic risk groups could not predict overall survival, however a trend was noted (*P = 0.093*).

**Figure 2:**
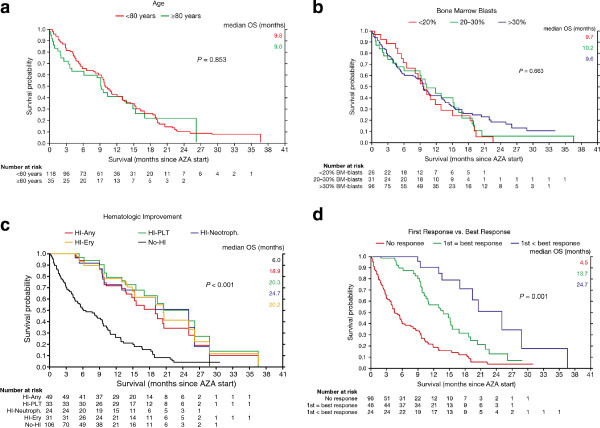
**Effect of various factors on overall survival (OS).** (**a**) Effect of Age on OS. (**b**) Effect of bone marrow blast percentage on OS. (**c**) Effect of achievement of any type of hematologic response on OS. (**d**) Effect of deepening of response after first response on OS. Patients experiencing a further deepening of response after first response had significantly longer overall survival, than those patients for whom first response was best response.

Baseline factors that did not significantly affect OS in univariate analyses (Additional file 1: Table S4) include WBC ≤/>10G/l (*P = 0.346*), LDH ≤/>225 IU/l (*P = 0.123*), S-EPO level (*P = 0.661*), RBC-TD (*P = 0.542*), and PLT-TD (*P = 0.149*). Age had no adverse effect on outcome of AML-patients treated with azacitidine, irrespective of whether a cut-off of </≥75 (*P = 0.174*) or </≥ 80 years was chosen (*P = 0.853*) (Figure 2a). Bone marrow blast count ≤30/>30% had no adverse effect on OS, irrespective of whether the whole cohort (*P = 0.663*) was analyzed, or whether patients with prior intensive CTX (*P = 0.313*), or all pretreated patients were excluded (*P = 0.127*) (Figure 2b, Additional file 1: Table S4). Prior treatment with ESA (*P = 0.873*), G-CSF (*P = 0.841*), low-dose cytarabine (*P = 0.630*) or one or several lines of conventional chemotherapy (*P = 0.113*) had no adverse effect on OS (Additional file 1: Table S4). In concordance, patients, for whom absence of CR or refractoriness to prior intensive chemotherapy was the reason for treatment with azacitidine, did not have worse OS (*P = 0.268*).

In multivariate analysis the following response related factors had a significant effect on overall survival: hematologic improvement (*P = 0.0001*) and marrow response (*P = 0.0005*) (Figure 1 and Additional file 1: Table S5). Any kind of response resulted in significantly longer OS, be it marrow response (24.7 vs. 15.2 vs. 2.3 months for CR vs. mSD vs. PD), achievement of PLT-TI (13.7 vs. 8.9 months), RBC-TI (19.3 vs. 9.6 months), or HI in one or all cell lineages (18.9 vs. 6.0 months) (Figures 1 and 2c, Additional file 1: Table S5). Continued azacitidine beyond first response resulted in further deepening of response in 34.3% of responders, which translated into significantly longer OS (24.7 months), compared with patients for whom first response was best response (13.7 months) (*P < 0.001*) (Figure 2d, Additional file 1: Table S5).

In multivariate analysis the occurrence of fatigue (*P = 0.0299*) or hematologic toxicity (*P = 0.0447*) as adverse events, had an independent prognostic effect on OS (Figure 1 and Additional file 1: Table S5). Azacitidine dose or schedule and the occurrence and duration of various non-hematologic adverse events had no negative impact on OS (Additional file 1: Table S4).

## Discussion

With 155 AML-patients included in the AAR and 8.4 million inhabitants, this is the highest per capita coverage of AML-patients in a nationwide registry, suggesting limited selection. The per capita inclusion of AML-patients into our national registry (18.9 per million inhabitants) is the largest as compared to data from other countries that has so far been published (0.06-6.58 per million inhabitants) (Additional file 1: Table S1) [15,17,21-26]. The number of AML diagnoses per year, as well as the number of AML-patients included in the respective year for Austria in general and Salzburg in particular, is presented in Additional file 1: Table S2 (data obtained from Statistics Austria, personal communication 14.03.13). The percentage of patients in the MRC cytogenetic high-risk category is 17% in our AML cohort, which seems less than the proportion of cytogenetic high-risk AML-patients reported in several, but not all previously published smaller AML-cohorts treated with azacitidine (Additional file 1: Table S1). Cumulating evidence suggests that patients with high-risk cytogenetics respond poorly to conventional chemotherapeutic approaches, and that these patients especially may be candidates for hypomethylating treatment. Therefore, according to our understanding, patients with high-risk cytogenetics should be overrepresented, -rather than underrepresented-, in underrepresented-, in our registry, if an explicit selection bias concerning cytogenetic risk categories were present.

Median age, age range, pretreatment cytogenetic risk groups, azacitidine schedule and median azacitidine cycles are comparable to those documented in much smaller AML-patient-subsets (n = 20-82) in almost all previously published clinical trials and observational data so far (Additional file 1: Table S1) Therefore we believe that selection bias is minimized, both with respect to the choice of AML-patients for azacitidine treatment, as well as for the decision to include the patients in the registry (Additional file 2: Figure S2A).

The most common hematologic and non-hematologic adverse events, and the rates thereof are almost identical to previously published data from controlled trials as well as retrospective analyses [16,27]. CR/mCR-, as well as HI-rates are comparable to, -and PR-rates slightly higher than-, the rates obtained in smaller series (n = 20-82) of AML-patients treated with azacitidine by others [15,17,21-25]. Due to higher PR-rates, the overall response rate (Table 2) is one of the highest documented for AML-patients treated with azacitidine so far.

Median OS of elderly AML-patients is still low, irrespective of treatment modality. Conventional chemotherapy and other treatment options such as low-dose cytarabine, topotecan, gemtuzumab ozogamicin, clofarabine, cloretazine, tipifarnib, or laromustine show median OS rates of 2.0-6.7 months [7-13,28-31]. In comparison, AML-patients with 20-30% blasts (formerly RAEB-t) treated with BSC only (n = 223; median age 73a) had a median OS of 5.2 months in a recent update of the Düsseldorf MDS registry data (U.Germing, personal communication, 17/09/2012). This is similar to 5.0 months OS of AML-patients randomized to best supportive care or low-dose cytarabine in a recent phase-III trial of untreated AML [32]. Therapy-naïve AML-patients (n = 242) randomized to decitabine in the same trial had a median OS of 7.7 months, which led to a positive opinion by EMA, with formal approval expected soon [32]. Previous reports of much smaller series of AML-patients treated with azacitidine documented median OS rates of 3, 7–9, 2.5-15, 8, 6–16 and 8.8 months, respectively [21-25,30]. (The reports giving OS-ranges did not separate the OS of AML- and MDS-patients explicitly). In light of the above, our OS results of 9.8 months are encouraging. Only two subanalysis of randomized clinical trials (CALGB-9211 (n = 27), AZA-001 (n = 55)) reported longer OS than observed in our cohort [17,33]. The authors of the latter stated that this is likely due to exclusion of patients with an estimated life expectancy <3 months, ECOG-PS >2, t-AML, prior treatment, or planned allogeneic SCT [16,17]. Overall, 61% of our registry population would have been excluded from this trial due to these reasons alone; if BM-blast count >30% is also taken into account, 90% would have been excluded. We thus consider our OS data to be remarkable, particularly since they were observed in a real-life AML-patient cohort, not selected or restricted by stringent inclusion/exclusion-criteria, and in which most patients had no other treatment option than azacitidine.

Significantly longer OS was observed in patients achieving any kind of response, be it marrow response, achievement of RBC/PLT-TI, or HI (Figures 1 and 2c, Additional file 1: Table S5). Similar results have been shown in smaller AML-patient cohorts (n = 11) treated with azacitidine [34]. Others, who did not analyze the HI-rate in their AML-cohorts (n = 25-55, BM-blasts 20-30%), also noted, that azacitidine can provide survival benefit, despite a low CR-rate [17], a finding which is similar to that seen in high-risk MDS-patients [16]. However, it is for the first time shown in multivariate analysis, that response in terms of HI alone, without requirement of concomitant BM-blast reduction, seems sufficient to confer longer OS in AML-patients treated with azacitidine (18.9 vs. 6.0 months) (Figure 1). If the commonly used AML response criteria [20] were to be applied, patients who experience HI without simultaneous BM-blast reduction would be considered as having neither CR nor PR, and thus as non-responders, with the result that treatment with azacitidine would/might be discontinued. In our opinion these patients should be considered as responders, and should continue to receive azacitidine until unambiguous clinical progression (i.e. renewed transfusion dependence and/or stark reduction of general performance).

We show for the first time in AML-patients treated with azacitidine, that further deepening of response after first response, i.e. achievement of BM-blast reduction in terms of PR or (m)CR after HI, seems to translate into significantly longer OS, compared with patients for whom first response was best response (Figures 1 and 2d, Additional file 1: Table S5). Similar results have been shown for MDS-patients treated with azacitidine [16,35]. Therefore, in analogy to recommendations for MDS [35,36], these data support the continued use of azacitidine in responding AML-patients.

It is possible, that a larger percentage of AML-MRF and t-AML may be present in our registry-population (Table 1). However, very little recent data is available on the incidence of all WHO-AML-subgroups in adults in Europe [37], and the recently published phase-III trial comparing decitabine vs. conventional care regimen also had a high percentage (35%) of secondary-AML [30]. In any case, t-AML is known to be associated with dismal prognosis [38], and AML-patients with preceding MDS/MPN and/or MDS-related cytogenetics (MRC) were shown to have worse OS than AML-NOS [39].

Our registry population includes many elderly and very old patients: 22% aged 75–79, 15% aged 80–84 and 8% ≥ 85 years, respectively; many of these very old patients would previously have been treated with best supportive care only. Importantly, age had no adverse effect on OS, irrespective of whether a cut-off of </≥75 (Additional file 1: Table S4) or </≥80 years was chosen (Figure 2a). In addition, only 40% of patients were untreated prior to azacitidine, whereas 39%, 7% and 6% were pretreated with conventional chemotherapy, low-dose cytarabine or ‘imids’, respectively (Table 1). In light of all of the above, we consider our response and OS results particularly relevant.

The absolute number of comorbidities had no adverse effect on OS. Accordingly, the HCT-CI [40], which is based on the number and weighting of comorbidities, could not discriminate patient-groups with different OS (Additional file 1: Table S4), whereas the ECOG-PS [41], which is based on the patient’s ability to cope with activities of everyday life, could (Figure 1, Additional file 1: Table S5). In our opinion, high age and presence of comorbidities should not lead to a decision to withhold treatment of azacitidine in favor of BSC, if the patient has an ECOG-PS < 2.

Azacitidine seems effective in AML, irrespective of BM-blast count (Additional file 1: Table S4, Figure 2b). Lack of correlation between survival and median pre-treatment BM-blast count has previously been reported in WHO-AML-patients (n = 40) [21]. We believe, that AML-patients with >30% BM-blasts should not be precluded from treatment with azacitidine.

Interestingly, patients experiencing hematologic toxicity grade 3–4 in any, one, several, or all cell lineages had significantly better OS (Figure 1). In clinical practice it is important to note that AML-patients may develop a worsening of cytopenia(s) during the first two months prior to azacitidine response, but this does not seem to be associated with an increased rate of infection or hemorrhage [14,15]. In fact, even when occurring, these events did not negatively impact OS in our cohort, and neither did non-hematologic toxicity, nor duration of adverse events (Additional file 1: Table S4). The majority of grade 3-4 hematologic adverse events were documented during early treatment cycles, suggesting that (a) patient tolerance to azacitidine increases as treatment continues, (b) a certain amount of aplasia-induction seems necessary before response occurs, and (c) likely reflects the fact that most patients respond after cycle 3. Patients in whom azacitidine dose had to be reduced due to an adverse event even had improved OS in univariate analyses. This effect was however lost in multivariate analysis. It seems fair to state that dose reductions were possible without negative impact on OS. We deduct that the occurrence of adverse events should not lead to permanent treatment discontinuation with azacitidine in most cases. Azacitidine should be continued as planned whenever possible, if necessary with dose reduction or treatment pause.

Achievement of the FDA-approved target-dose of 75 mg/m^2^×7 days did not have a significant effect on OS, and neither did the predominantly applied schedule (Additional file 1: Table S4). Similar results have been shown by others for MDS [27,40]. Concerning OS of responding patients only (n = 70), it made no relevant difference which regimen or dose was used in the first cycle, or which regimen or dose was predominantly used over all cycles (Additional file 1: Table S4). The 5-2-2 regimen and/or dose reductions, be it in days (d1-5) and/or dose per day (100 mg flat), seem feasible, safe, and without loss of efficacy. In spite of the impression of non-inferiority of alternative schedules and dosages demonstrated by our data, we still recommend initiation of azacitidine in the FDA-approved dosage and regimen.

We acknowledge the limitations of data obtained from a registry and are fully aware, that clinical trials may not be substituted, but only complemented by these. If adequate in size and analyzed carefully, registry data can be of value in supplementing or extending data from trials. The strength of this manuscript lies in the size of the documented patient group, that allows statistical generation of hypothesis, which may be assessed by clinical trials in due time. It was our explicit intention and the design of the registry not to select patients, but in contrast to obtain a widespread view of the use, toxicity and effects of azacitidine in a real-world clinical setting. We believe this is substantiated by the following facts:

a) The population was substantially pretreated;

b) 48 patients (31%) received only one or two cycles of azacitidine;

c) 90% of our patient cohort would have been excluded from the pivotal AZA-001 trial due to reasons listed in the discussion of the text;

d) azacitidine was used as last resort, or last line therapy in at least a part of the cohort with a very low life expectancy; these patients would have received best supportive care only before azacitidine was available;

e) 40% of patients were treated with azacitidine 1^st^ line, as we believed this to be an appropriate option given their disease characteristics, patient profile and in the absence of clinically superior alternatives.

We moderately hypothesize and suggest, that our rather ‘aggressive’ initiation of treatment, irrespective of comorbidities, bone marrow blast count, age or pre-treatment, as well as the ‘persistent’ continuation of treatment with azacitidine once the drug was commenced (i.e. treatment until overt clinical progression, rather than mere laboratory signs of progression such as stable or slightly rising blast counts in blood or bone marrow) might play a part in our results, although this cannot directly be proven by the data as presented.

## Conclusions

In conclusion, azacitidine seems to be well tolerated and efficacious in WHO-AML-patients in a real-world non-trial clinical setting. The observed median OS of 9.8 months in this largely pretreated cohort including 66% of patients with bone marrow blasts >30% is encouraging. We confirm the previously reported non-essentiallity of CR for prolongation of OS in our cohort of AML-patients treated with azacitidine. In our opinion, age >80, number of comorbidities, and/or bone marrow blasts >30% should not preclude patients from treatment with this drug, which should be continued as long as response is durable, and onset of overt clinical progression occurs.

## Methods

### Registry design and patient eligibility

WHO-classified AML [42] and treatment with at least one dosage of azacitidine were the sole inclusion criteria (Additional file 2: Figure S2A). No other formal inclusion/exclusion criteria existed. Treatment indication and the decision to offer treatment with azacitidine, as well as dosage, dose reductions/escalations, application route, and administration schedule were exclusively based on the risk/benefit-estimation of the treating physician.

Registry design and timelines can be taken from Additional file 3: Figure S2B. Patients receiving azacitidine prior to EMA-approval, as well as patients with >30% BM-blasts were informed of off-label use and gave written informed consent to treatment with azacitidine. Informed consent to allow the collection of personal data was obtained for all retrospectively documented patients who were alive, as well as for all prospectively included patients.

### Data collection and monitoring

Data was entered by physicians and/or trained clinical trial personnel at the respective centers. Central monitoring of all data entered in the eCRF including response evaluations was performed as a quality control measure by LP, in order to assure data integrity and plausibility (cut-off date 21.01.2012). Missing data are low and documented as such. Centers received queries specifying incomplete data or questions to reconfirm data, if necessary.

### Assessment of efficacy, safety and endpoints

Bone marrow punctures/aspirations were performed as clinically necessary; Marrow response, hematologic improvement and overall response were assessed according to current IWG-criteria [19,20]. Central monitoring of response in each cycle was performed. A total of 97% of our cohort had either AML with >30% bone marrow blasts, was refractory to prior conventional chemotherapy or had AML with 20-30% bone marrow blasts. The remaining 3% of patients were refractory to other therapies (e.g. low-dose cytarabine). We therefore are certain, that the patients included truly suffered from AML, even without central review of the blast count.

Toxicity and adverse events were assessed according to the NCI Toxicity Criteria (http://ctep.cancer.gov/protocolDevelopment/electronic_applications/ctc.htm) and Common Terminology Criteria for AE (CTCAEv.4) (http://evs.nci.nih.gov/ftp1/CTCAE/About.html). Grade 3–4 cytopenias reported, are those that were documented as adverse events, and thus felt to be a worsening of pre-existing cytopenia by the respective treating physicians.

### Statistical analysis

Overall survival was assessed using the Kaplan-Meier method. Univariate analyses were performed with log-rank tests. Cox-regression stratified on the various factors was used for univariate analyses of risk-factors for OS. For multivariate analysis Cox-regression with stepwise selection (entry-level 0.25; level for keeping the variable 0.15) was used. Univariate analyses were performed and confirmed by two independent statisticians [H.A., B.R.]. The confirmed results were the basis for multivariate analysis. All variables with *P < .05* in univariate analyses were included in multivariate analysis, except for those cases, where parsimony would have been disrupted due to redundancy in the variables. All analyses were performed with SPSS and SAS. No adjustments were made for multiple testing.

## Competing interests

*Consultant or advisory role:* Lisa Pleyer, Celgene, Bristol-Myers Squibb, Novartis; Sonja Burgstaller, Celgene; Michael Pfeilstöcker, Celgene, Novartis; Michael Girschikofsky, Mundipharma; Alois Lang, Celgene; Hubert Angermann, Unidata Geodesign GmbH; Reinhard Stauder, Celgene; Alexander Egle, Celgene; Richard Greil, Bristol-Myers-Squibb, Cephalon, Celgene;

*Honoraria:* Lisa Pleyer, Celgene, Bristol-Myers Squibb, Novartis, AOP Orphan Pharmaceuticals; Thomas Melchardt, Mundipharma; Sonja Burgstaller, Mundipharma, Novartis, AOP Orphan Pharmaceuticals; Michael Pfeilstöcker, Celgene, Novartis; Michael Girschikofsky, Pfizer, Mundipharma; Reinhard Stauder, Ratiopharm, Celgene; Richard Greil, Amgen, Eisai, Mundipharma, Merck, Janssen-Cilag, Genentech, Novartis, Astra-Zeneca, Cephalon, Boehringer-Ingelheim, Pfizer, Roche, Bristol-Myers Squibb, Sanofi Aventis; Peter Krippl, Roche, Amgen, Pfizer, Mundipharma, Galxo Smith Klein, PharmaMar, Astra Zeneca; Alexander Egle, Celgene;

*Research funding:* Michael Girschikofsky, Pfizer; Reinhard Stauder, Ratiopharm, Novartis, Celgene; Richard Greil, GSK, Amgen, Genentech, Ratiopharm, Celgene, Pfizer, Mundipharma, Cephalon; Peter Krippl, Pfizer, Roche; Alexander Egle, Celgene;

*Expert testimony:* Peter Krippl, Amgen, Roche, Mundipharma;

*Other remuneration:* Thomas Melchardt, travel support: Amgen, Sanofi Aventis, Roche, Celgene, BMS, Janssen-Cilag, Böhringer Ingelheim; Reinhard Stauder and Martina Mitrovic were supported by Verein Senioren-Krebshilfe;

## Authors’ contributions

All authors had access to all the clinical data, and were kept up-to date with recent results of the registry via oral presentations from LP at regular intervals. All authors participated in regular critical discussions concerning the status and direction of the registry as well as the data to be published. All authors had the opportunity to review the final manuscript prior to submission. The primary and corresponding authors had final responsibility for the decision to submit for publication. *Conception and design:* LP, RG; *Statistics and online CRF:* HA, LP; *Collection and assembly of data:* LP, RS, SB, MS, CT, MP, SS, TM, MM, MG, AL, PK, TS, AE, WL, DV, HA, RG; *Data analysis and interpretation:* LP, RG; *Manuscript writing:* LP; *Critical revision of the paper:* LP, RG, AE, RS, SB, MS, CT, MP, SS, TM, MM, MG, AL, PK, TS, WL, DV, HA; *Final approval of Manuscript:* LP, RS, SB, MS, CT, MP, SS, TM, MM, MG, AL, PK, TS, AE, WL, DV, HA, RG; Provision of patients: LP, RS, SB, MS, CT, MP, MG, AL, PK, TS, AE, WL, DV, RG.

## Supplementary Material

Additional file 1: Table S1 Comparison of overall response rates of all current full publications on AML patients treated with azacitidine. **Table S2.** Number of AML diagnoses per year in Austria, and patient recruitement to the Austrian Azacitidine Registry (AAR). **Table S3.** Azacitidine treatment schedule. **Table S4.** Factors that did not significantly affect overall survival. **Table S5.** Factors significantly influencing overall survival. Click here for file

Additional file 2: Figure S1 (CONSORT-Diagram A Describes the design of, and patient eligibility for the Austrian Azacitidine Registry (AAR). Click here for file

Additional file 3: Figure S1 (CONSORT-Diagram B. Describes the timelines of the Austrian Azacitidine Registry (AAR). Click here for file
