# Supplementary material for: Azacitidine in patients with WHO-defined AML – Results of 155 patients from the Austrian Azacitidine Registry of the AGMT-Study Group
Source: J Hematol Oncol. 2013 Apr 29;6:32. doi: 10.1186/1756-8722-6-32 (PMC3655844; doi:10.1186/1756-8722-6-32)
Supplement: Additional file 1: Table S1 — Comparison of overall response rates of all current full publications on AML patients treated with azacitidine. Table S2. Number of AML diagnoses per year in Austria, and patient recruitement to the Austrian Azacitidine Registry (AAR). Table S3. Azacitidine treatment schedule. Table S4. Factors that did not significantly affect overall survival. Table S5. Factors significantly influencing overall survival. [file 1756-8722-6-32-S1.docx]

**Supplemental** **Table 1. Comparison of overall response rates of all current full publications on AML patients treated with AZA (Blood)**

| **Variable** | **Germany^21^** | **Italian^22^** | **Dutch^25^** | **AZA 001^17^** | **CALGB**  **8421^15^** | **CALBG**  **8921^15^** | **CALGB**  **9221^15^** | **French^24^** | **Pennsylvania^23^** | **Swiss**  **^26^** | **AAR** |
| --- | --- | --- | --- | --- | --- | --- | --- | --- | --- | --- | --- |
| n | 40 | 82 | 31 | 55 | 25 | 26 | 27 | 26 | 20 | 52 | 155 |
| Inhabitants, mio | **81.7** | **60.6** | **16.7** | **897.3**^#^ | **311.6** | **311.6** | **311.6** | **65.4** | **12.7** | **7.9** | **8.2** |
| n AML-pts. treated with AZA/capita | **0.49** | **1.36** | **1.86** | **0.06** | **0.08** | **0.08** | **0.09** | **0.40** | **1.57** | **6.58** | **18.9** |
| Phase | I/II | Retrosp. | Retrosp. | III-subanal. | I/II-subanal. | II-subanal. | III-subanal. | Retrosp. | Retrosp. | Retrosp. | Retrosp. |
| Median age (range) | 72 (32–84) | 77 (46–87) | 70 (40–84) | 70 (52–80) | 65 (35–81) | 66 (23–82) | 69 (31–92) | 69 (37–89) | 69 (44–80) |  | 73 (33–91) |
| AZA schedule^*^ | d1–5 | d1–7 | d1–7 | d1–7 | d1–7 | d1–7 | d1–7 | d1–7 | d1–7 |  | d1-5 (16%)  d1-7 (57%)  5-2-2 (22%) |
| Median cycles | 3 (0–16) | 4 (1–22) | 5 (1–19) | 8 (1–39) | *ND* | *ND* | *ND* | 6 (1–28) | *ND* | 6 (3-20) ¥ | 4 (1–24) |
| Median FU, months | 13 | 12 | 8 | 20 | *ND* | *ND* | *ND* | 20 | *ND* | 12.2 | 7.7 |
| Median OS, months | 3 | 7–9 | 6–16 | 24.5 | *ND* | *ND* | 19.3 | 8 | 2.5–15 | 8.6 | 9.8 |
| ORR, %  CR/(m)CR  PR  HI | 30  5  8  18 | 36  20  12  34 | 39  23  3  13 | n.g.  18  n.g.  n.g. | 48  12  4  32 | 35  12  0  23 | 37  7  0  30 | >=39  16  23  n.g. | 60  20  25  15 | >=31  13  4  31 | 45  13  21  9 |
| BM blasts < 30%  > 30% | 42  *ND* | 35  54 | WHO-AML  *ND* | 23  0 | WHO-AML  *ND* | WHO-AML  *ND* | WHO-AML  *ND* | MPD-AML  65 | *ND*  29 | 17¥  83 | 37  63 |
| Cytogenetics, % | Int: 70†  High: 30 | Int: 37†  High: 23 | Int: 68‡  High: 32 | Int: 69‡  High: 26 | *ND* | *ND* | *ND* | Int: 38§  High: 46 | Int: 60¶  High: 20 | Int: 64¥£  High: 29 | Int: 74†  High: 17 |

AAR indicates Austrian Azacitidine Registry; Subanal., subanalysis; AZA, azacitidine; retrosp, retrospective; FU, follow up; mo, months; OS, overall survival; ORR, overall response rate; CR, complete response; mCR, marrow complete response; PR, partial response; HI, hematologic improvement; *ND*, not determined; BM, bone marrow; WHO, World Health Organization; Int., intermediate; MPD, myeloproliferative disease;

^#^108 participating centers from 15 countries (http://clinicaltrials.gov/ct2/show/study/NCT00071799?show_locs=Y#locn)

^*^75 mg/m^2^ except for the Italian study:**^22^** 42% 100 mg flat;

†MRC-criteria, MRC cytogenetic risk groups, Medical research Council cytogenetic risk groups

‡ISCN criteria, International System for Cytogenetic Nomenclature

§IPSS cytogenetic risk criteria, IPSS cytogenetic risk groups, International Prognostic Scoring Index cytogenetic risk groups

¶Merely defined as normal, simple and complex cytogenetic abnormalities

¥Reported for only a subset of patients (n=38)

£HOVON classification

**Supplemental Table 2. Number of AML diagnoses per year in Austria, and patient recruitement to the AAR**

|  | Statistiks Austria | Data from the AAR | | | Data from Tumor Registry Salzburg | Data from AAR |
| --- | --- | --- | --- | --- | --- | --- |
|  | n AML diagnoses  in Austria | n AZA start  in Austria† | n entered in eCRF  in Austria | n centers  in Austria | n AML diagnoses  in Salzburg | n AZA start  in Salzburg† |
| 2007 | 324 | 10 | n.a. | 3 | 26 | 4 |
| 2008 | 280 | 23 | n.a. | 6 | 33 | 13 |
| 2009 | 275 | 47 | 56 | 9 | 20 | 14 |
| 2010 | 303 | 44 | 49 | 10 | 26 | 14 |
| 2011 | n.g. | 31 | 50 | 7 | 29 | 9 |
| Total | 1182 | 155 | 155 | 12 | 134 | 54 |

AAR indicates Austrian Azacitidine Registry; AZA, azacitidine; eCRF, electronic case report form; n.g., not given; n.a.

†Please note, that the patients started on azacitidine in a respective year, are not necessarily those diagnosed in the same year.

Supplemental Table 2 shows the number of AML new diagnoses per year in Austria. In addition, the table shows the numbers of AAR-patients started on azacitidine per respective year, the number of centers including patients in the AAR per respective year, as well as the number of patients included in the AAR per respective year (note: as ethics committee approval was obtained 01.02.2009, data entry commenced as of this time-point). The respective numbers for the center of Salzburg only are also shown.

**Supplemental Table 3. Azacitidine treatment schedule**

| **Variable** | **n pts. ^*^, (%)** | **Mean dose**†**, mg** | **Median dose**†**, mg** | **Dose range**†**, mg** |
| --- | --- | --- | --- | --- |
| **AZA schedule all patients**  AZA 1–5  AZA 1–7  AZA 5-2-2  AZA others | 24 (15.5)  89 (57.4)  34 (21.9)  8 (5.2) | 675  886  823  695 | 685  924  900  700 | 375-1000  385-1155  385-1188  130-1260 |
| **AZA schedule responders**  AZA 1–5  AZA 1–7  AZA 5-2-2  AZA others | 10 (14.1)  37 (52.1)  21 (29.6)  3 (4.2) | 733  871  837  641 | 754  912  897  600 | 375-955  437-1054  455-1113  575-748 |
| **AZA schedule non-responders**  AZA 1–5  AZA 1–7  AZA 5-2-2  AZA others | 14 (16.7)  52 (61.9)  13 (15.5)  5 (6.0) | 648  924  782  489 | 643  924  796  405 | 350-1000  700-1155  487-1000  489-900 |

AZA indicates azacitidine;

**^*^**Refers to patients who predominantly had this type of azacitidine schedule

†Refers to total given azacitidine cycles

**Supplemental Table 4. Factors that did not significantly affect overall survival**

| **Factors not affecting OS** | **n** | **Median OS, mo*** | ***P* value**† |  | **Factors not affecting OS** | **n** | **Median OS, mo*** | ***P* value**† |
| --- | --- | --- | --- | --- | --- | --- | --- | --- |
| **Age**  < 80 years  ≥ 80 years | 119  36 | 9.8  9.5 | 0.853 |  | **MRC cytogenetic risk**  Good  Intermediate  High | 3  114  26 | 8.1  10.8  8.4 | 0.093 |
| **Age**  < 75 years  ≥ 75 years | 85  70 | 9.4  10.2 | 0.174 |  | **Infectious complications**  None  Grade 1–4 | 57  98 | 9.4  10.3 | 0.070 |
| **WBC count**  Non-MP-AML (≤ 10 G/l)  MP-AML (> 10 G/l) | 122  33 | 9.8  9.4 | 0.346 |  | **Febrile neutropenia**  No  Yes | 127  28 | 9.4  14.6 | 0.384 |
| **LDH**  ≤ 225 U/l  > 225U/l | 68  81 | 11.8  8.1 | 0.123 |  | **Bleeding events**  No  Yes | 141  14 | 9.7  10.9 | 0.758 |
| **Serum EPO (IU/l)**  < 50  50 < 500  > 500 | 18  22  3 | 4.5  9.1  8.4 | 0.661 |  | **Non-hematologic toxicity**  None  Grade 1–2  Grade 3–4 | 113  27  15 | 9.5  10.8  13.7 | 0.549 |
| **BM blasts total cohort**  < 20%  20–30%  > 30% (off label use) | 26  31  98 | 9.6  1ß.2  9.6 | 0.663 |  | **GIT-Toxicity**  None  Grade 1–2 | 122  33 | 9.5  12.9 | 0.284 |
| **BM blasts de novo AML**  20–30%  > 30% (off label use) | 19  44 | 10.2  14.6 | 0.127 |  | **Surgery**  None  Elective  Emergency | 137  11  7 | 9.5  18.9  15.2 | 0.160 |
| **BM blasts excluding prior conv. CTX**  20–30%  > 30% (off label use) | 26  66 | 10.2  12.8 | 0.313 |  | **Fall**  With fracture  With hemorrhage  No fall | 14  8  5  109 | 11.8  9.8  -  10.3 | 0.408 |
| **Prior intensive CTX**  None  ≥ 1 line of CTX | 95  60 | 10.5  7.6 | 0.113 |  | **Pain**  No pain + mild pain  Moderate + severe pain | 135  20 | 9.7  9.8 | 0.857 |
| **Reason for treatment**  No CR to CTX/allo-SCT  Other reasons | 50  101 | 5.7  10.8 | 0.268 |  | **Injection site reaction**  None  Erythema or mild soreness  Pain/inflammation/phlebitis | 126  23  6 | 9.5  10.8  15.4 | 0.198 |
| **Prior trt. with ESA**  No  Yes | 138  15 | **9.8**  10.9 | 0.873 |  | **AE duration**  < 3days  < 1week  1 < 2 weeks  2 < 3weeks  3 < 4weeks  ≥ 4weeks | 36  21  20  16  11  51 | 5.7  12.8  9.5  10.2  10.9  12.9 | 0.164 |
| **Prior trt. with G-CSF**  No  Yes | 133  19 | 9.7  10.2 | 0.841 |  | **Target dose‡**  < target dose  ≥ target dose | 70  85 | 10.8  8.9 | 0.154 |
| **Prior trt. with LD-Ara-C**  No  Yes | 145  10 | 9.6  15.0 | 0.722 |  |  |  |  |  |
| **RBC-TD prior to AZA**  No  Yes | 58  97 | 10.8  9.6 | 0.542 |  | **Predominant schedule (all pts.)**  d1–5  d1–7 or 5-2-2 | 24  123 | 12.6  10.2 | 0.912 |
| **PLT-TD prior to AZA**  No  Yes | 95  60 | 11.8  9.4 | 0.149 |  | **Schedule in 1^st^ cycle (responders)**  d1–5  d1–7  5-2-2  others | 8  46  9  8 | 16.8  15.0  15.0  18.5 | 0.828 |
| **HCT-CI**  Low risk  Intermediate risk  High risk | 46  46  50 | 10.2  9.0  9.7 | 0.207 |  | **Predominant schedule (responders)**  d1–5  d1–7  5-2-2  others | 10  37  21  3 | 21.7  14.6  15.0  18.5 | 0.224 |
| **Number of comorbidities**  < 3  ≥ 3 | 103  52 | 9.8  9.0 | 0.151 |  | **Dose in 1^st^ cycle (responders)**  < 600 mg  601–800 mg  > 800 mg | 4  14  53 | 16.8  15.4  15.0 | 0.939 |
| **Number of comorbidities**  < 4  ≥ 4 | 127  28 | 10.2  5.3 | 0.086 |  | **Predominant dose/cycle (responders)**  < 600 mg  601–800 mg  > 800 mg | 6  19  46 | 18.5  15.2  14.6 | 0.650 |

OS indicates overall survival; mo, months; MP, myeloproliferative; TD, transfusion dependence; MRC cytogenetic risk groups, Medical research Council cytogenetic risk groups; HCT-CI, Hematopoietic Stem Cell Transplant Comorbidity Index; trt, treatment; CTX, chemotherapy; GIT, gastro-intestinal tract; AE, adverse event;

^*^1 day = 0.0328549112 months;

† Log-Rank (Mantel-Cox);

‡Target dose = 75 mg/m^2^ x 7 +/- 10%

**Supplemental Table 5. Factors significantly influencing overall survival**

|  | | | **Univariate Analysis** | | | **Multivariate Analysis** | | | | |
| --- | --- | --- | --- | --- | --- | --- | --- | --- | --- | --- |
| **Variable** | **n** | **Median OS, mo*** | ***P* value**† | **HR** | **95% CI** | ***P* value**† | | **HR** | | **95% CI** |
| **PB-blasts**  0%  > 0% | 49  97 | 15.0  8.9 | 0.01 | 2.113 | 1.371–3.257 | 0.0398 | | 0.592 | | 0.360-0.976 |
| **Prior ‘imids’**  No  Yes | 146  9 | 9.7  3.0 | 0.08 | 2.425 | 1.225-4.800 | *ND* due to few pt. numbers in the ‘Yes’ category; | | | | |
| **ECOG-PS**  ECOG < 2  ECOG ≥ 2 | 110  40 | 10.9  8.1 | 0.001 | 1.930 | 1.285–2.898 | 0.0397 | | 0.619 | | 0.392-0.978 |
| **RBC-TI**  No  Yes | 33  36 | 9.6  19.3 | < 0.001 | 0.316 | 0.168–0.595 | *ND* due to redundancy of the variable with ‘hematologic improvement’; | | | | |
| **PLT-TI**  No  Yes | 19  24 | 8.9  13.7 | 0.01 | 0.296 | 0.142–0.612 | *ND* due to redundancy of the variable with ‘hematologic improvement’; | | | | |
| **Hematologic improvement**  no HI  HI-any | 106  49 | 6.0  18.9 | < 0.001 | 0.756 | 0.676–0.844 | 0.0001 | 0.367 | | 0.219–0.614 | |
| **Best marrow response - ITT**  CR (response for MVA)  mCR (response for MVA)  PR (response for MVA)  mSD (non-response for MVA)  PD (non-response for MVA) | 15  5  32  19  4 | 24.7  25.8  13.5  15.2  2.3 | < 0.001 | 0.429 | 0.219-0.839 | 0.0005 | 0.379 | | 0.220–0.653 | |
| **Best overall response**  OR-yes  OR-no | 70  85 | 15.2  4.6 | < 0.001 | 3.223 | 2.198–4.724 | *ND* due to redundancy of the variable with ‘Best marrow response’ and ‘Hematologic improval’; | | | | |
| **Response deepening**  No response  1^st^ response = best response  1^st^ response < best response | 85  46  24 | 4.6  13.7  24.7 | < 0.001 | 0.389 | 0.289–0.523 | *ND* due to redundancy of the variable with ‘Best marrow response’ and ‘Hematologic improval’; | | | | |
| **Time to best response**  ≤ 4 months  > 4 months | 34  36 | 10.8  29.3 | 0.001 | 0.348 | 0.184–0.661 | *ND* due to redundancy of the variable with ‘Best marrow response’ and ‘Hematologic improval’ | | | | |
| **IPSS cytogenetic risk**  Good  Intermediate  Poor | 90  26  28 | 10.8  9.4  9.1 | 0.027 | 1.394 | 1.087–1.787 | 0.6430 (did not meet 0.25 criterium for model entry); | | | | |
| **Adverse karyotype**  Yes (-7q, -7, abn(3q), complex)  No (other aberrations, normal) | 28  115 | 5.1  10.5 | 0.009 | 0.543 | 0.342-0.862 | *ND* due to redundancy of the variable with ‘IPSS cytogenetic risk’ | | | | |
| **Fatigue**  None  Relieved by rest  Not relieved by rest  Limiting self care | 99  17  23  16 | 9.8  13.5  13.7  4.2 | < 0.0001 | 0.316 | 0.181-0.552 | 0.0299 | 0.522 | | 0.290–0.939 | |
| **Hematologic toxicity G3**–**4**  Yes  Thrombocytopenia G3–4  Neutropenia G3–4  Anemia G3–4  No | 70  39  50  33  85 | 13.7  10.9  13.5  13.7  8.1 | 0.0022 | 0.551 | 0.376-0.806 | 0.0447 | 0.655 | | 0.433-0.990 | |
| **Does reduction due to AE**  Yes  No | 28  127 | 15.0  9.6 | 0.016 | 0.556 | 0.342-0.902 | 0.8085 (did not meet 0.25 criterium for model entry); | | | | |

d indicates days; mo, months; HR, hazard ratio; CI, confidence interval; PB, peripheral blood; *ND*, not done; ECOG-PS, Eastern Cooperative Oncology Group Performance Score; TI, transfusion independence; HI, hematologic improvement; mSD, marrow stable disease; PD, progressive disease; OR, overall response; IPSS, international prognostic scoring system;

^*^1 day = 0.0328549112 months;

†Log-Rank (Mantel-Cox);
