# Supplementary material for: Azacitidine in patients with WHO-defined AML – Results of 155 patients from the Austrian Azacitidine Registry of the AGMT-Study Group
Source: J Hematol Oncol. 2013 Apr 29;6:32. doi: 10.1186/1756-8722-6-32 (PMC3655844; doi:10.1186/1756-8722-6-32)
Supplement: Additional file 2: Figure S1 — (CONSORT-Diagram A Describes the design of, and patient eligibility for the Austrian Azacitidine Registry (AAR). [file 1756-8722-6-32-S2.pptx]

## Slide 1
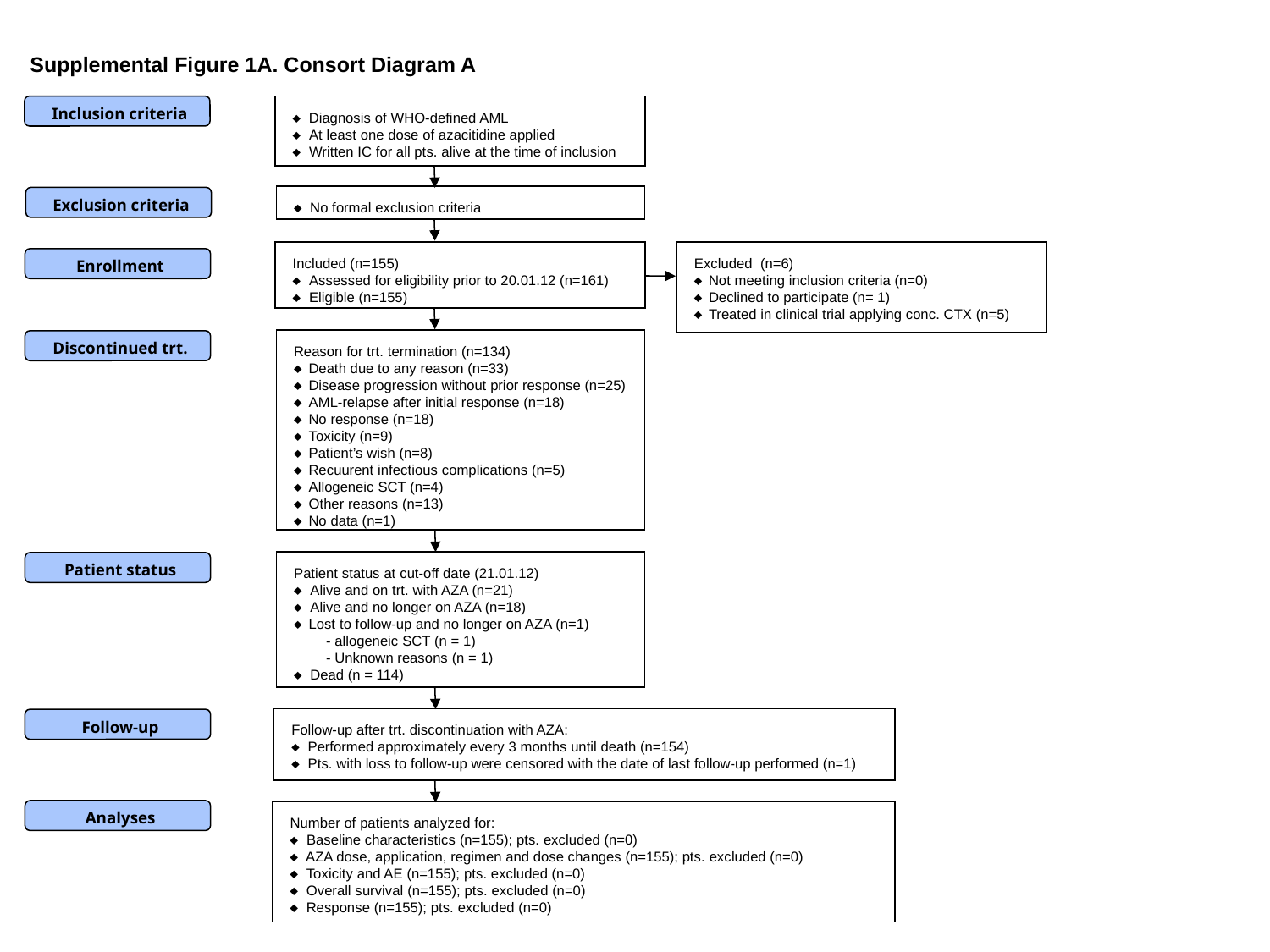

Supplemental Figure 1A. Consort Diagram A
Inclusion criteria
¨ Diagnosis of WHO-defined AML
¨ At least one dose of azacitidine applied
¨ Written IC for all pts. alive at the time of inclusion
¨ No formal exclusion criteria
Exclusion criteria
Included (n=155)
¨ Assessed for eligibility prior to 20.01.12 (n=161)
¨ Eligible (n=155)
Excluded (n=6)
¨ Not meeting inclusion criteria (n=0)
¨ Declined to participate (n= 1)
¨ Treated in clinical trial applying conc. CTX (n=5)
Enrollment
Reason for trt. termination (n=134)
¨ Death due to any reason (n=33)
¨ Disease progression without prior response (n=25)
¨ AML-relapse after initial response (n=18)
¨ No response (n=18)
¨ Toxicity (n=9)
¨ Patient’s wish (n=8)
¨ Recuurent infectious complications (n=5)
¨ Allogeneic SCT (n=4)
¨ Other reasons (n=13)
¨ No data (n=1)
Discontinued trt.
Patient status at cut-off date (21.01.12)
¨ Alive and on trt. with AZA (n=21)
¨ Alive and no longer on AZA (n=18)
¨ Lost to follow-up and no longer on AZA (n=1)
 - allogeneic SCT (n = 1)
 - Unknown reasons (n = 1)
¨ Dead (n = 114)
Patient status
Follow-up after trt. discontinuation with AZA:
¨ Performed approximately every 3 months until death (n=154)
¨ Pts. with loss to follow-up were censored with the date of last follow-up performed (n=1)
Follow-up
Analyses
Number of patients analyzed for:
¨ Baseline characteristics (n=155); pts. excluded (n=0)
¨ AZA dose, application, regimen and dose changes (n=155); pts. excluded (n=0)
¨ Toxicity and AE (n=155); pts. excluded (n=0)
¨ Overall survival (n=155); pts. excluded (n=0)
¨ Response (n=155); pts. excluded (n=0)
