# Supplementary material for: Azacitidine in patients with WHO-defined AML – Results of 155 patients from the Austrian Azacitidine Registry of the AGMT-Study Group
Source: J Hematol Oncol. 2013 Apr 29;6:32. doi: 10.1186/1756-8722-6-32 (PMC3655844; doi:10.1186/1756-8722-6-32)
Supplement: Additional file 3: Figure S1 — (CONSORT-Diagram B. Describes the timelines of the Austrian Azacitidine Registry (AAR). [file 1756-8722-6-32-S3.pptx]

## Slide 1
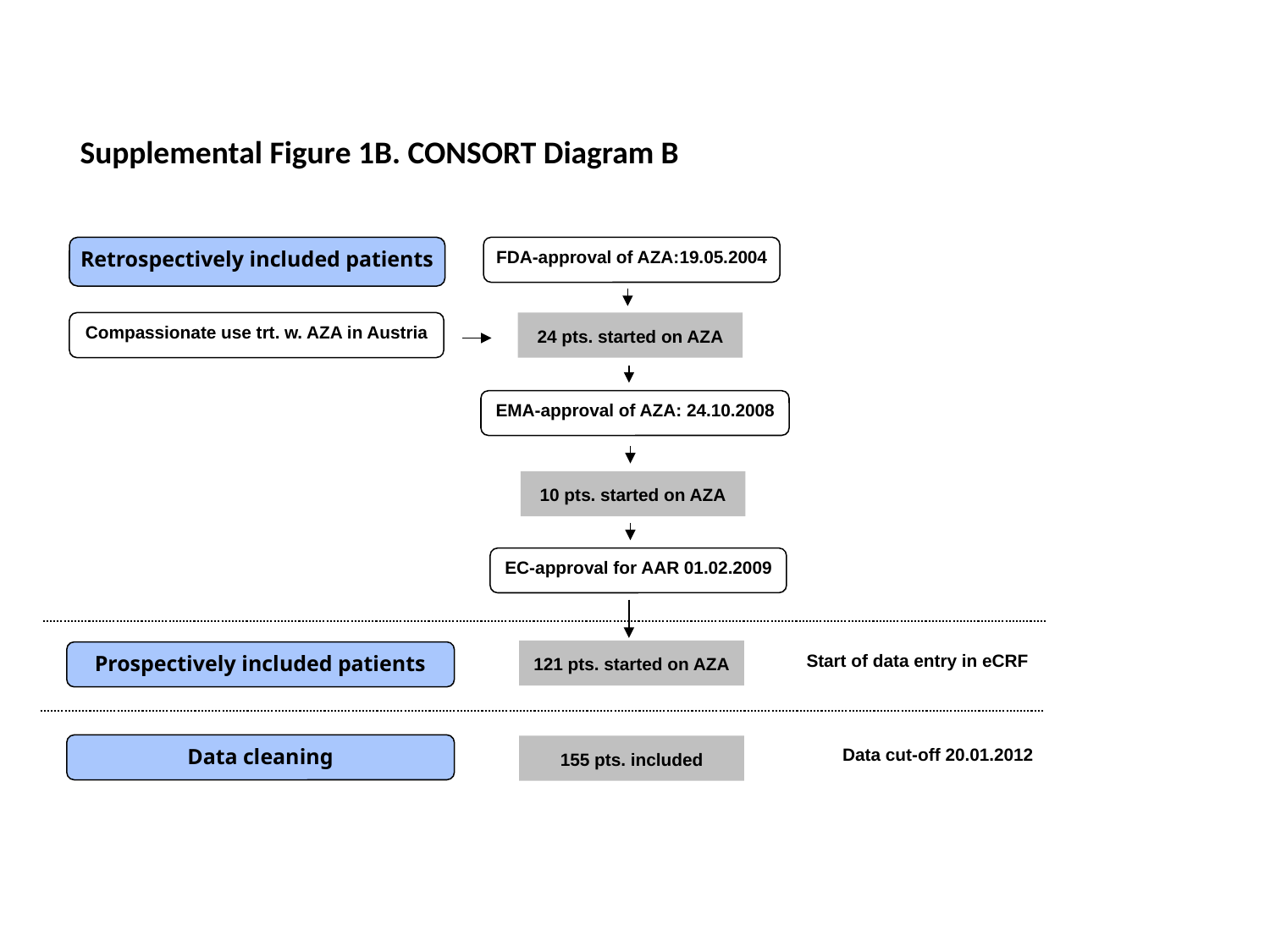

Supplemental Figure 1B. CONSORT Diagram B
Retrospectively included patients
FDA-approval of AZA:19.05.2004
Compassionate use trt. w. AZA in Austria
24 pts. started on AZA
EMA-approval of AZA: 24.10.2008
10 pts. started on AZA
EC-approval for AAR 01.02.2009
Start of data entry in eCRF
121 pts. started on AZA
Prospectively included patients
Data cut-off 20.01.2012
Data cleaning
155 pts. included
